# Correlation Between Sarcopenia and Growth Rate of the Future Liver Remnant After Portal Vein Embolization in Patients with Colorectal Liver Metastases

**DOI:** 10.1007/s00270-020-02416-6

**Published:** 2020-01-23

**Authors:** M. Schulze-Hagen, D. Truhn, F. Duong, S. Keil, F. Pedersoli, C. K. Kuhl, G. Lurje, U. Neumann, P. Isfort, P. Bruners, M. Zimmermann

**Affiliations:** 1 Department of Diagnostic and Interventional Radiology, RWTH Aachen University Hospital, Aachen, DE Germany; 2 Institute of Imaging and Computer Vision, RWTH Aachen University, Aachen, DE Germany; 3 Department of Surgery and Transplantation, RWTH Aachen University Hospital, Aachen, DE Germany

**Keywords:** Portal vein embolization, Sarcopenia, Liver hypertrophy, FLR, PVE

## Abstract

**Purpose:**

To investigate whether sarcopenia and myosteatosis correlate with the degree of hypertrophy (DH) and kinetic growth rate (KiGR) of the future liver remnant (FLR) in patients with colorectal liver metastases undergoing portal vein embolization (PVE) in preparation for right hepatectomy.

**Materials and Methods:**

Forty-two patients were included. Total liver volume and FLR volume were measured before and 2–4 weeks after PVE. KiGR of the FLR was calculated. Sarcopenia was assessed using the total psoas muscle volume (PMV), the psoas muscle cross-sectional area (PMCS) and the total skeletal muscle index (L3SMI) at the level of 3rd lumbar vertebra. Degree of myosteatosis was assessed by mean muscle attenuation at L3 (L3MA). Correlations between muscle indices and DH and KiGR were assessed using simple linear regression analyses.

**Results:**

Mean DH was 8.9 ± 5.7%, and mean KiGR was 3.6 ± 2.3. Mean PMV was 55.56 ± 14.19 cm^3^/m^3^, mean PMCS was 8.76 ± 2.3 cm^2^/m^2^, mean L3SMI was 45.6 ± 9.89 cm^2^/m^2^, and mean L3MA was 27.9 ± 18.6 HU. There was a strong positive correlation between PMV and DH (*R* = 0.503, *p* = 0.001) and PMV and KiGR (*R* = 0.545, *p* < 0.001). Furthermore, there was a moderate correlation between PMCS and KiGR (*R* = 0.389, *p* = 0.014). L3SMI and L3MA were neither associated with DH (*p* = 0.390 and *p* = 0.768, respectively) nor with KiGR (*p* = 0.188 and *p* = 0.929, respectively).

**Conclusion:**

We identified a positive correlation between PMV and PMCS, as markers for sarcopenia, and the KiGR of the FLR after PVE. PMV and PMCS might therefore aid to identify patients who are poor candidates for FLR augmentation using PVE alone.

**Electronic supplementary material:**

The online version of this article (10.1007/s00270-020-02416-6) contains supplementary material, which is available to authorized users.

## Introduction

Portal vein embolization (PVE) of the right-sided branches of the portal vein is nowadays a routine procedure for patients undergoing (extended) right hemihepatectomy in order to induce hypertrophy of the left liver lobe/future liver remnant (FLR) and prevent postoperative liver insufficiency [1, 2]. Depending on various factors, such as the degree of liver cirrhosis and pretreatment with hepatotoxic chemotherapy, the volume of the FLR should be at least 20–40% of the total liver volume in order to be eligible for surgery (TLV) [3–5]. The degree of hypertrophy (DH) of the FLR after PVE also varies greatly between patients, and several studies have developed approaches to predict FLR growth based on laboratory values and clinicopathologic data, such as bilirubin levels and history of previous chemotherapy or liver surgery [6, 7].

Recently, sarcopenia, the degenerative loss of skeletal muscle mass and function [8], has been shown to correlate with morbidity and mortality after major liver surgery and has been shown to adversely affect the outcome in various other severe diseases as well [9–12]. Most notably, it has also been reported to predict survival in patients with colorectal liver metastases undergoing radioembolization [13]. Besides general loss of muscle mass, the loss of muscle function is often associated with increased proportions of inter- and intramuscular fat, which is called myosteatosis and represents a subelement of sarcopenia [14]. Myosteatosis has been identified as another important parameter and has also been linked to adverse outcomes in a variety of different diseases such as, e.g., liver cirrhosis and patients undergoing liver surgery [15–17].

Measurements of the psoas major muscle on cross-sectional imaging are increasingly used to assess sarcopenia and myosteatosis, as they can be performed in routine CT scans. These opportunistic imaging markers are easily accessible and can potentially be exploited to predict survival and the course of certain diseases in individual patients [18].

Since sarcopenia and myosteatosis often times indicate patient frailty and thus a decreased physiologic reserve capacity of a patient, it can be hypothesized that these entities correlate with poor FLR hypertrophy after PVE. The purpose of this study was therefore to investigate whether different markers of sarcopenia, i.e., total psoas muscle volume (PMV), psoas muscle cross-sectional area (PMCS) as well as total skeletal muscle mass (L3SMI) at the L3 level, and myosteatosis, i.e., the mean muscle attenuation at the L3 level, correlate with the DH and KiGr of the FLR in patients with colorectal liver metastases undergoing PVE in preparation for subsequent right-sided hepatectomy.

## Materials and Methods

Approval for this retrospective study was waived by the institutional review board (IRB). For this study, patients with colorectal liver metastases who underwent portal vein embolization of liver segments V–VIII in preparation for right hepatic lobectomy between 05/2012 and 05/2018 were retrospectively identified from the institutional radiology database (*n* = 70).

Inclusion criteria for this study were the presence of a venous CT scan of the whole abdomen at least 4 weeks before PVE for measurement of muscle indices and baseline liver volumes and presence of a venous CT scan of the liver 1–5 weeks after PVE for measurement of post-interventional liver volumes. Only patients in whom both examinations were available and conducted according to a standardized protocol—as specified below—were included. Patients with incomplete CT examinations and imaging or laboratory evidence of liver cirrhosis were excluded.

CT imaging data were acquired according to a standardized protocol on multi-slice CT scanners (Somatom Definition Flash and Somatom Definition AS, Siemens Medical Systems, Forchheim, Germany) at our institution. CT scan parameters were as follows: tube voltage 120 kV, section collimation 128 mm and pitch 0.6. Tube current was modulated according to Siemens CareDose4D. Portal venous phase was conducted with a delay of 75 s using a body weight adapted dose (1 ml per kg bodyweight) of iodinated, nonionic contrast agent (Ultravist 300, Bayer AG, Leverkusen, Germany). Patient demographics and data concerning treatment before PVE, regarding surgical treatment and postoperative follow-up, were extracted from the electronic medical records.

### Muscle Indices

All muscle indices were measured using 5 mm slices of the baseline CT scans (venous phase) before PVE. Muscle indices were measured using two different software tools:

Measurements of PMV were taken automatically using a dedicated machine learning algorithm. The algorithm was developed in cooperation with the Institute of Imaging and Computer Vision of the RWTH Aachen University. It uses a 3D atlas segmentation approach in combination with a generative adversarial network architecture-based (GAN type) convolutional neural network. Training of the algorithm was conducted on an independent dataset, which was manually segmented by an abdominal radiologist, using 68 single psoas major muscle segmentations in CT scans in venous phase with a slice thickness of 5 mm. Volumetric results were given in cm^3^ and divided by the patient’s height in m^3^. A video of the automated segmentation process is available as supplementary material online. Finally, all of the 42 automatically generated segmentations were reviewed by a consultant radiologist.

**Fig. 1:**
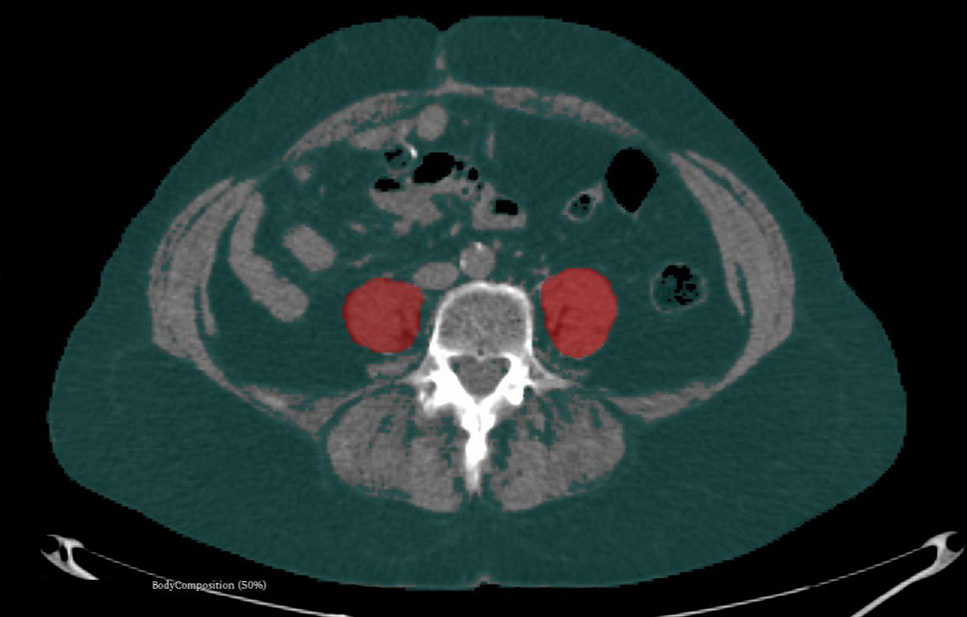
Sample case of semiautomatic segmentation of the psoas major muscle cross-sectional area (PMCS). Using the 3D slicer software, the cross-sectional area of both psoas muscles (red overlay) was measured on the axial slice with the largest muscle diameter

**Fig. 2:**
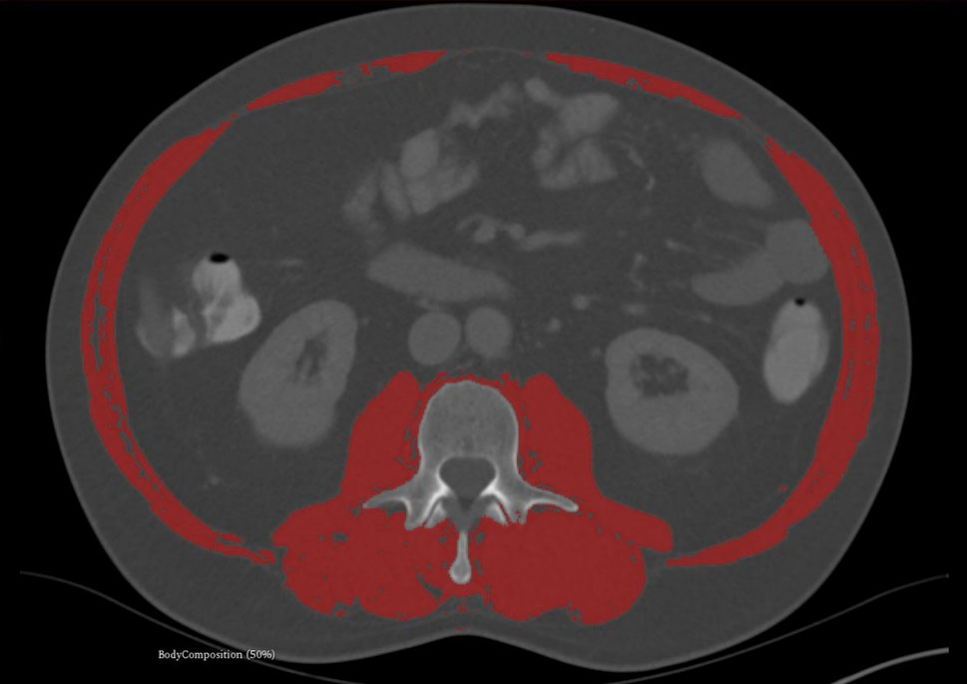
Sample case of a semiautomatic segmentation of the skeletal muscle index at the level of the 3rd lumbar vertebra (L3SMI). The L3SMI, which comprises all muscles with a red overlay (i.e., psoas muscles, paravertebral muscles and abdominal muscles) at the L3 level, was measured using the 3D slicer software

Measurements of the PMCS, L3SMI and L3MA were taken manually using a semiautomatically segmentation tool, 3D slicer, which is an open source software platform for medical image informatics (https://www.slicer.org/) [19]. PMCS was measured on the CT slice with the largest psoas muscle diameter in the axial plane. L3SMI as well as the L3MA was measured at the center plane of the 3rd lumbar vertebra on axial CT slices. Examples of the segmentations of PMCS and L3SMI are shown in Figs. 1 and 2.

### Portal Vein Embolization

Informed consent was obtained from all patients before the intervention, and all procedures were performed by interventional radiologists with over 5 years of experience. All PVE procedures were performed via an ipsilateral approach. Using ultrasound guidance, a right hepatic portal vein radicle was punctured and a 6-French-Sheath was advanced into the main portal vein. Subsequently, the portal vein branches of liver segments V–VIII were gradually embolized under fluoroscopic guidance using a 3:1 mixture of *n*-butyl-2-cyanoacrylate (Histoacryl, Braun, Germany) and iodized oil (Lipiodol, Guerbet, France). After successful embolization of all right hepatic portal vein branches up until about 2–3 cm from the main portal vein division, all catheters and the sheath were removed and the puncture tract was sealed by administration of the same mixture during removal of the sheath.

### Liver Volumetry and Calculation of Markers for FLR Hypertrophy

TLV and volumes of the right and left hepatic lobes were measured on contrast-enhanced CT scans with 5 mm slice thickness before and after PVE using a dedicated software tool (Liver, Philips Intellispace; Philips, Eindhoven, Netherlands). The left hepatic lobe consisting of segments II, III and IV was considered the FLR. From these results, we calculated the following FLR hypertrophy metrics:

- degree of hypertrophy (DH), the relative increase of the percentage of the FLR of the TLV: (Vol_FLR Post_ % Vol_TLV Post_) − (Vol_FLR Pre_ % Vol_TLV Pre_)
- kinetic growth rate (KIGR): KIGR = DH (%)/time elapsed since PVE in weeks.

### Statistics

Separate simple linear regression analyses were performed with PMV, PMCS, L3SMI and L3MA as independent variables and the DH and KiGR of the FLR as dependent variables. A *p* value < 0.05 was considered statistically significant for all tests performed. The effect size was classified as small (*R* ≥ 0.1), medium (*R* ≥ 0.3) and large (*R* ≥ 0.5) according to Cohen [20]. Continuous variables were summarized using proportions, mean, median and standard deviation. All statistical analyses were done using SPSS statistical software (version 25; IBM, Armonk, NY, USA).

## Results

### Patient Cohort

A total of 42 patients (32 males, 10 females) with a mean age of 62.5 ± 9.5 years were included in this study. According to the body mass index (BMI), the majority of patients (32/42, 76%) had a normal weight or were slightly overweight (BMI between 18.5 and 30 kg/m^2^). One patient (1/42, 2%) was underweight (BMI < 18.5 kg/m^2^), seven patients (7/42, 17%) had class I obesity (BMI between 30 and 35 kg/m^2^) and two patients (2/42, 5%) had class II obesity (BMI between 35 and 40 kg/m^2^). Laboratory markers of liver function, i.e., serum bilirubin and INR, were within normal limits in all but one patient, in whom serum bilirubin levels were mildly elevated (33 μmol/l) but who had a normal INR. The mean time between the PVE and the follow-up CT for assessment of hypertrophy of the FLR was 2.9 ± 0.9 weeks (range 0.9–5.1). Further patient demographics are summarized in Table 1.

**Table 1:** Patient demographics

Total number of patients	*n* = 42
Age (years)	62.9 ± 9.5 (range 39–78)
Male/female	32/10
Patients’ height	175 ± 8 cm
Patients’ weight	80 ± 15 kg
Body mass index	26.1 ± 4.7 kg/m^2^ (range 17.9–38.7 kg/m^2^)
Size of largest hepatic metastatic lesion	4.1 ± 2.5 cm (range 0.7–11.3 cm)
Number of hepatic metastases	4.5 ± 2.6 (range 1–13)
Preoperative serum bilirubin	8 ± 5 μmol/l (range 3–33 μmol/l)
Serum bilirubin on postoperative day 5–7	26 ± 42 μmol/l (range 5–239 μmol/l)
Preoperative INR	1.02 ± 0.08 (range 0.87–1.15)
INR on postoperative day 5–7	1.17 ± 0.27 (range 0.91–2.7)

### Liver Volumetrics and Growth of the FLR

Mean TLV before PVE was 1657 ± 408 ml, and mean TLV after PVE was 1768 ± 456 ml. Mean absolute volume of the FLR before and after the intervention was 570 ± 206 ml and 759 ± 245 ml, respectively, which translates into a mean volume increase in the FLR of 188 ± 97 ml. The mean percentage of the FLR of the TLV before the PVE was 34.3 ± 8.3% and after PVE was 43.2 ± 9.6%, and thus the mean DH was 8.9 ± 5.7%. The calculated mean KiGR was therefore 3.6 ± 2.3.

### Measurements of Muscle Indices

The mean PMV was 55.56 ± 14.19 cm^3^/m^3^, the mean PMCS was 8.76 ± 2.3 cm^2^/m^2^, the mean L3SMI was 45.6 ± 9.89 cm^2^/m^2^, and the mean L3MA was 27.9 ± 18.6 HU.

### Association Between KiGR and Muscle Indices

**Fig. 3:**
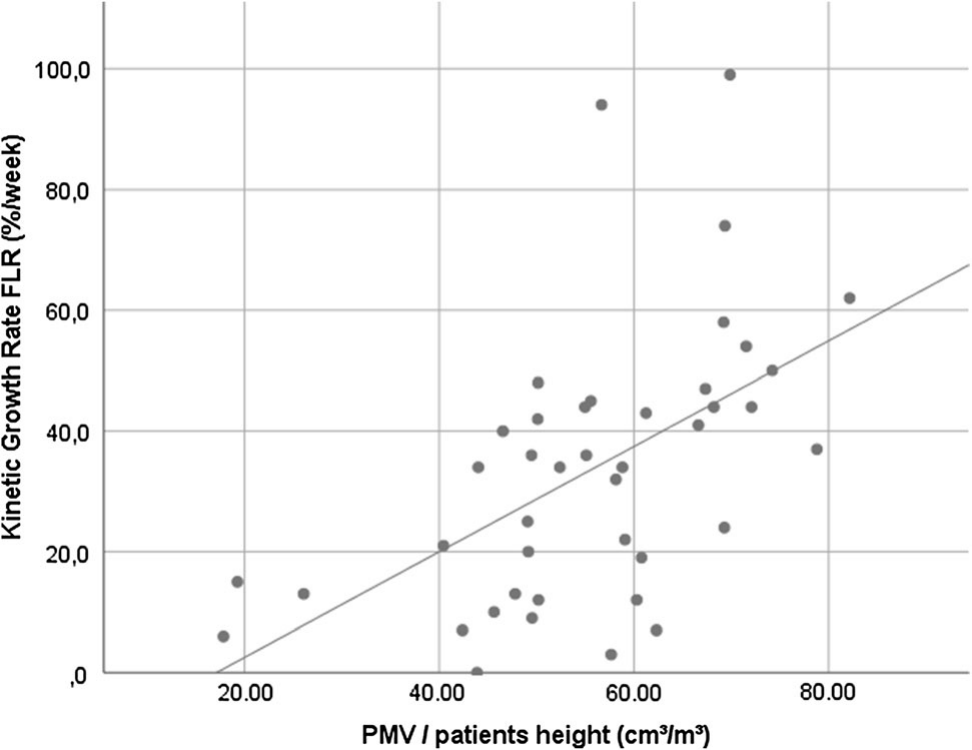
The scatter plot demonstrates the correlation between total psoas muscle volume (PMV) and the kinetic growth rate of the FLR (KiGR) (*R* = 0.545, *p* < 0.001)

In the linear regression analyses, the PMV was statistically significantly associated with the KiGR of the FLR (*R* = 0. 545, *R*^2^ = 0.297, *p* < 0.001) (Fig. 3), which is classified as a strong effect size according to Cohen. The full equation for this linear regression model was as follows: KiGR = −14.944 + 0.873 * PMV.

**Fig. 4:**
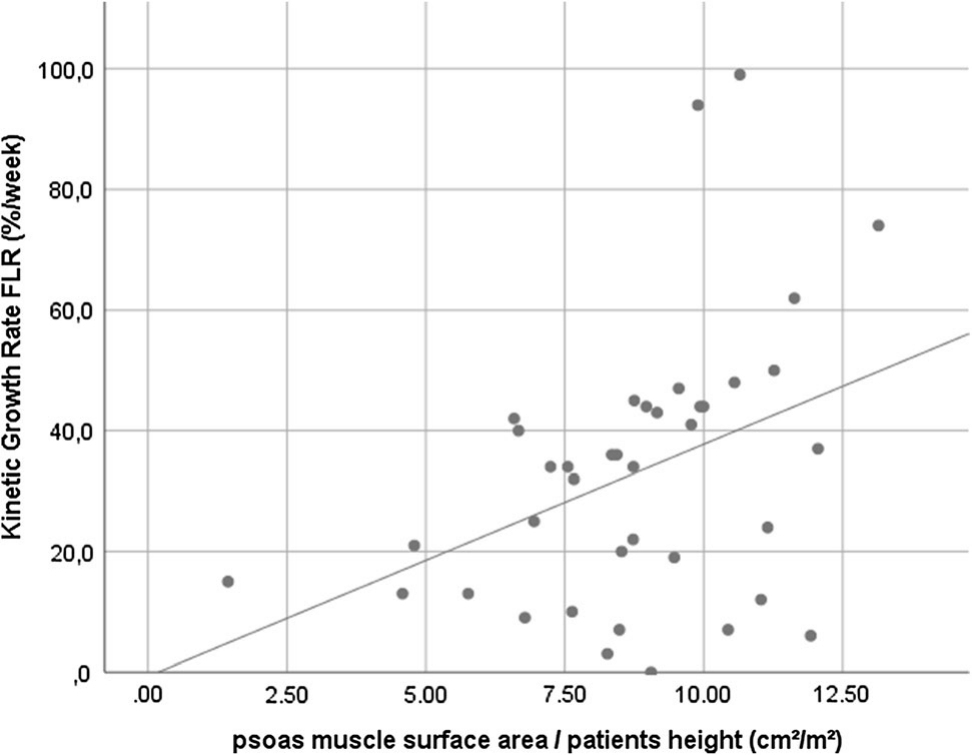
This scatter plot shows the correlation between psoas muscle cross-sectional area (PMCS) and the kinetic growth rate of the FLR (KiGR) (*R* = 0.389, *p* = 0.014)

The correlation between the PMCS and the mean KiGR was much lower, but still moderate (*R* = 0. 389, *R*^2^ = 0.151, *p* = 0.014) (Fig. 4). The full equation for this linear regression model was as follows: KiGR = −0.708 + 3.846 * PMCS. The L3SMI (*p* = 0.188) and the L3MA (*p* = 0.929) were not statistically significantly associated with the KiGR.

### Association Between Degree of Hypertrophy (DH) and Muscle Indices

There was a statistically significant correlation between the PMV and DH of the FLR (*R* = 0.503, *R*^2^ = 0.253, *p* = 0.001). The full equation for this linear regression model was as follows: DH = −24.214 + 2.031 * PMV. On the other hand, PMCS (*p* = 0.085), L3SMI (*p* = 0.390) and L3MA (*p* = 0.768) were not statistically significantly associated with DH.

### Surgical Treatment

All patients developed sufficient hypertrophy of the FLR (≥ 30% of the TLV or ≥ 40% in patients with extensive history of chemotherapy) after PVE and were thus considered technically resectable. Eventually, 38 out of the 42 patients successfully underwent (extended) right hemihepatectomy after a mean of 42 ± 39 days after PVE. In the remaining four patients, the planned resection was canceled due to intraoperatively discovered extrahepatic metastases (*n* = 3) and due to intraoperatively newly discovered metastases in the FLR (*n* = 1).

According to the 50/50 criteria [21], 37/38 resected patients had a sufficient hepatic function postoperatively. One of the 38 resected patients had a complicated postoperative course, however, including an NSTEMI on postoperative day 2, and he subsequently developed hepatic insufficiency with an increase in serum bilirubin levels to 239 μmol/l and INR to 2.7 on postoperative day 5. He ultimately died of multi-organ failure 11 days after surgery.

## Discussion

In this study, we demonstrate that a reduced psoas major muscle mass—as an indicator of sarcopenia—has a significant impact on the hypertrophy of the future liver remnant in patients undergoing portal vein embolization: there is a strong positive correlation between the PMV and to a lesser extent also the PMCS and the KiGR of the FLR patients with colorectal liver metastases undergoing PVE.

DH and particularly the KiGR of the FLR have been identified as the most important markers for postoperative morbidity and mortality in patients undergoing major liver resection after PVE [22, 23]. Since time for hypertrophy of the FLR is highly variable, there is a need for reliable predictors of FLR growth in order to minimize wait time between PVE and surgery depending on the individual patient. Several experimental and clinical studies have demonstrated a significant association between portal vein embolization and post-embolization intrahepatic tumor progression in patients with colorectal liver metastases [24–27]. Most importantly, development of new metastases in the future liver remnant after PVE can profoundly impact the further management of patients or even result in some patients becoming unresectable [25, 28]. It is therefore critical to keep the time between PVE and surgery as short as possible while at the same time allowing sufficient hypertrophy of the FLR to avoid postsurgical liver insufficiency. Furthermore, identifying patients with predictably slow FLR growth before PVE would allow for modification of the individual treatment plan: these patients could for example undergo total venous deprivation in addition to PVE to further enhance the KiGR of the FLR [29] or could be scheduled for a few cycles of neoadjuvant chemotherapy between PVE and hepatectomy to allow more time for FLR growth while keeping tumor growth under control at the same time.

Several different imaging markers for assessment of sarcopenia have been used in the past, and so far there is no consensus on which one of these markers is the best and should be used routinely. In this study, we investigated three different markers for sarcopenia with very different results. The L3SMI and the PMCS are two of the most commonly used markers for sarcopenia and therefore initially seemed to be the logical choice for investigating whether sarcopenia affects growth of the FLR. However, we found that the L3SMI had no statistically significant impact on the KiGR of the FLR, while the PMCS showed a significant correlation with the KiGR of the FLR. Since previous studies have suggested that single slice areas might be inaccurate compared to segmentations of the full psoas major muscle [30], we decided to proceed with measurements of the total psoas muscle volume for further investigation and found a strong positive correlation between the PMV and the KiGR, i.e., patients with a larger psoas volume had a higher KiGR rate of the FLR. Of course, the PMV requires three-dimensional measurements and is therefore significantly more time-consuming to acquire compared to the two-dimensional measurements for L3SMI and PMCS, at least when measurements are taken without a dedicated software tool. For the purpose of this study, a dedicated custom-made machine learning algorithm was therefore used for acquiring these PMV measurements.

Despite these interesting results, we do not advocate to use the PMV or cross-sectional areas as stand-alone factors to predict FLR growth in patients and plan treatment accordingly. Instead, it should be considered as complementary to other, previously identified predictors such as maximum serum bilirubin before PVE or prior chemotherapy [7].

There are several limitations to this study. First, this is a retrospective, single-center study. However, all data were prospectively acquired and all patients were treated according to a standardized protocol with respect to PVE, to surgical management and to follow-up. Second, the study cohort is small and therefore the statistical power of this analysis is limited which warrants further validation of the results of this study in a larger patient cohort. Third, the machine learning algorithm used for measurements of total psoas major volumes is not yet publicly available, which limits the practical applicability.

In conclusion, there is a positive correlation between the PMV and PMCS with the KIGR in patients with colorectal liver metastases undergoing PVE. In combination with other previously identified predictors, e.g., maximum serum bilirubin before PVE or a history of chemotherapy, CT-detectable sarcopenia could assist in stratifying patients according to their expected growth rate of the FLR, which in turn may guide the timeline for surgical treatment in individual patients.

## Electronic supplementary material

Below is the link to the electronic supplementary material.

Supplementary material 1 (GIF 8501 kb)

## Abbreviations

FLR: Future liver remnant
PVE: Portal vein embolization
TLV: Total liver volume
KiGR: Kinetic growth rate
DH: Degree of hypertrophy
PMV: Psoas muscle volume
PMCS: Psoas muscle cross-sectional area at the largest diameter
L3SMI: Skeletal muscle index at the level of the 3rd lumbar vertebra
L3MA: Mean muscle attenuation at the level of the 3rd lumbar vertebra
