# Supplementary figures and images for: Correlation Between Sarcopenia and Growth Rate of the Future Liver Remnant After Portal Vein Embolization in Patients with Colorectal Liver Metastases
Source: Cardiovasc Intervent Radiol. 2020 Jan 23;43(6):875–81. doi: 10.1007/s00270-020-02416-6 (PMC7225189; doi:10.1007/s00270-020-02416-6)

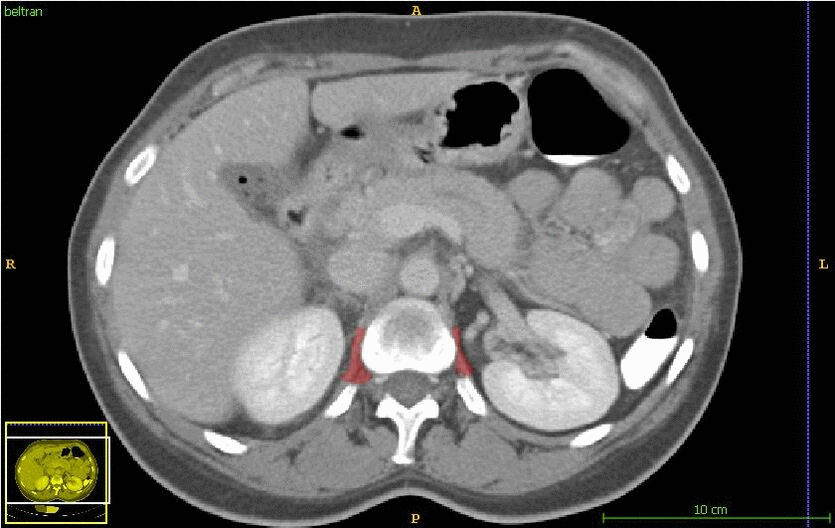

Supplement: Supplementary file 1 — Supplementary material 1 (GIF 8501 kb) [file 270_2020_2416_MOESM1_ESM.gif]
